# Crystal bending in triple-Laue X-ray interferometry. Part I. Theory

**DOI:** 10.1107/S1600576723002844

**Published:** 2023-05-12

**Authors:** C. P. Sasso, G. Mana, E. Massa

**Affiliations:** a INRIM, Istituto Nazionale di Ricerca Metrologica, Strada delle Cacce 91, 10135 Torino, Italy; b Dipartimento di Fisica, UNITO, Università di Torino, Via Pietro Giuria 1, 10125 Torino, Italy; Oak Ridge National Laboratory, USA; North Carolina State University, USA

**Keywords:** crystal X-ray interferometry, dynamical theory of X-ray diffraction, Laue diffraction, bent crystals

## Abstract

The measured value of the (220) lattice-plane spacing of silicon 28 using scanning X-ray interferometry is essential to realize the kilogram by counting ^28^Si atoms. An assumption made is that the measured lattice spacing is the bulk value of an unstrained crystal forming the analyser of the interferometer. However, analytical and numerical studies of the X-ray propagation in bent crystals suggest that the measured lattice spacing might refer to the analyser surface. To confirm the result of these studies and to support experimental investigations of the matter by phase-contrast topography, a comprehensive analytical model is given of the operation of a triple-Laue interferometer having the splitting or recombining crystal bent.

## Abstract 2

Previous studies suggested that the silicon lattice spacing measured by X-ray interferometry might refer to the crystal surface. To confirm this result and to support experimental investigations of the matter, this paper gives a comprehensive analytical model of the operation of a triple-Laue interferometer having one of the splitting and recombining crystals bent.

## 1. Introduction

Crystal X-ray interferometry splits and recombines X-rays while maintaining coherence. Monolithic interferometry was first demonstrated by Bonse & Hart (1965 ▸), and the first split-crystal interferometers for X-rays were operated in 1968 and 1969 (Bonse & te Kaat, 1968 ▸; Deslattes, 1969 ▸).

When the crystal recombining the interfering X-rays (the analyser) is separated, the interference signal is sensitive to movements orthogonal to the diffracting lattice planes. Since a displacement by one plane creates a 2π phase shift, such an interferometer allowed measurement of the lattice parameter of ^28^Si with parts per billion accuracy (Massa *et al.*, 2011 ▸, 2015 ▸). This result led to the determination of the Avogadro constant (Fujii *et al.*, 2018 ▸), the realization of the kilogram by counting atoms (Massa *et al.*, 2020*b* ▸) and the redefinition of the international system of units (SI) (Wiersma & Mana, 2021 ▸).

To realize the kilogram, an essential assumption is that the measured lattice spacing is the bulk value of the unstrained analyser (a blade, typically 1 mm thick). However, surface relaxation, reconstruction and oxidation might cause lattice strains (Melis *et al.*, 2015 ▸, 2016 ▸; Massa *et al.*, 2020*a* ▸). Furthermore, analytical and numerical studies of the X-ray propagation in a bent crystal (*e.g.* because of a difference between the surface stresses of the two surfaces) suggest that the measured lattice spacing might refer to the surface rather than to the bulk (Mana *et al.*, 2004*a* ▸,*b* ▸; Apolloni *et al.*, 2008 ▸).

To confirm the results of these studies and to support experimental tests of this prediction by phase-contrast topography, we give an analytical model of the operation of a triple-Laue interferometer having, one at a time, the splitter, mirror and analyser crystals cylindrically bent. Our interest is in the phase of the diffracted waves, rather than the intensity profile arising when using bent crystals *e.g.* to focus X-rays or as analysers for X-ray spectroscopy (Nesterets & Wilkins, 2008 ▸; Kaganer *et al.*, 2020 ▸; Qi *et al.*, 2021 ▸; Guigay & Sanchez del Rio, 2022 ▸).

This paper is organized as follows. The interferometer operation is outlined in Section 2. Sections 3 and 4 deal with the strain field in a cylindrically bent crystal, the reciprocal vector of the strained lattice and the description of the wavefields in perfect crystals as a two-state quantum system. In Section 5, we solve the Takagi–Taupin equations for X-ray propagation in a bent (symmetrically cut) crystal slab. The propagation in free space is examined in Section 6. Sections 7 and 8 deal with the wavefields leaving a bent crystal and a triple-Laue interferometer having the splitting or recombining crystal bent. In the conclusion, we outline predictions that have been verified by the phase-contrast topography of a monolithic interferometer having one of its crystals bent by a thin copper film (Massa *et al.*, 2023 ▸).

All the symbolic computations were carried out with the aid of *Mathematica* (Wolfram Research, 2021*a* ▸); the relevant notebook is given as supporting information. To view and interact with it, readers need to download the *Wolfram Player* which is free of charge (Wolfram Research, 2021*b* ▸).

## 2. Interferometer operation

**Figure 1:**
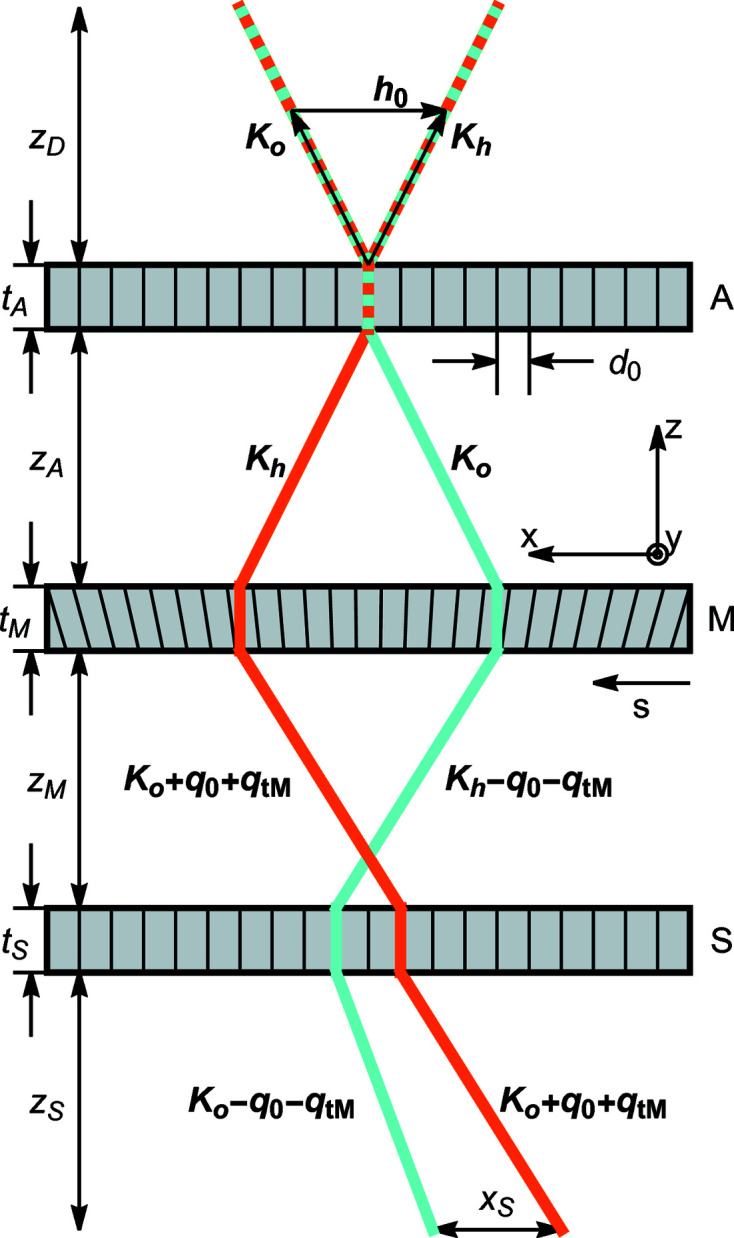
Top view of a symmetrically cut LLL interferometer having a bent mirror and operating in coplanar geometry. S splitter, M mirror, A analyser. The *z* axis is orthogonal to the crystal surfaces; the *x* axis is orthogonal to the diffracting planes. Orange and cyan indicate arms 1 and 2, respectively. The mirror bending makes the rays that leave the interferometer in the 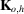 directions exit the source at different points and in different directions. An ideal geometry is assumed, 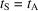 and 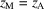 . 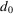 , spacing of the unstrained diffracting planes; 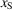 , separation at the source of the rays interfering collinearly.

Fig. 1 ▸ shows schematically a symmetrically cut triple-Laue (LLL) X-ray interferometer having a bent mirror and operating in coplanar geometry. It also gives the meaning of some of the symbols that we will use. The interferometer consists of three plane-parallel Si crystals, splitter, mirror and analyser, about 1 mm thick and cut in such a way that the diffracting {220} planes are perpendicular to the surfaces. They split and recombine 17 keV X-rays from a conventional Mo source.

To measure the spacing of the diffracting planes, the analyser is moved orthogonally to them. Owing to this displacement, the intensity of the forward-transmitted and reflected beams varies sinusoidally, the period being ideally equal to the sought spacing. The measurement result is the ratio between the displacement (measured absolutely via optical interferometry) and the number of X-ray fringes observed.

## 3. Strained crystals

We consider, one at a time, the interferometer crystals cylindrically bent about an axis perpendicular to the *x*–*z* plane (see Fig. 1 ▸) and approximate the *x* component of the displacement field, 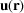 , by the hyperbolic paraboloid (Nesterets & Wilkins, 2008 ▸; Kaganer *et al.*, 2020 ▸) 

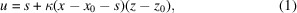

where 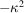 is the Gauss curvature and positive κ values equal downward bendings, as shown in Fig. 1 ▸ (Weisstein, 2023 ▸), 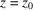 is the neutral plane, and 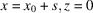 is the bending axis. Before bending, the input surface of the crystal is *z* = 0 and the output one *z* = *t*.

Equation (1) follows from the elastic theory of thin (isotropic) plates having thickness *t*, where 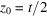 , but, for the sake of generality, we do not assume 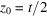 . The limit 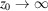 with 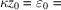 const. describes a crystal uniformly strained. The limit 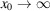 with 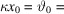 const. describes a crystal uniformly tilted. In general, in the case of thin crystals, (1) is the first-order approximation of any smooth displacement field.

Equation (1) is not strictly valid in the presence of anisotropy, unpaired surface stresses and Dirichlet boundary conditions imposed at the crystal base. Our finite element analyses and experimental verifications are given by Massa *et al.* (2023 ▸). In particular, we observed that a copper film coated on one of the surfaces bends the crystal in such a way that its opposite, naked, surface lies in the neutral, 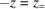 plane.

We introduced the overall crystal displacement *s* because, in the determination of the Si lattice parameter by a split-crystal interferometer, the analyser is moved along the *x* axis. In the analysis of this measurement, 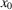 is contained in *s* and omitted from (1). In the phase-contrast topography of a monolithic interferometer, the *x* position of the X-ray beam is varied step by step. In the analysis of this measurement, 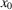 encodes the X-ray beam displacement and *s* is contained in 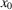 and omitted from (1).

Owing to the bending, the diffracting planes are rotated by 

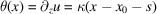

and strained by 

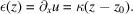

A positive 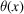 rotates the diffracting planes in the 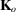 direction and a positive strain means a larger diffracting-plane spacing.

The electric susceptibility of the strained crystal (*e.g.* in Fig. 1 ▸, the mirror) is 

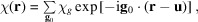

where 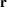 is a position vector and 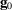 is a reciprocal vector of the unstrained crystals (*e.g.* in Fig. 1 ▸, of the splitter and analyser). By expanding 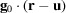 in series, we find that 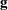 = 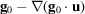 is a reciprocal vector of a locally perfect crystal.

Therefore, by using (1), the reciprocal vector of the strained diffracting planes is 

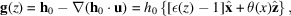

where 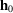 is the reciprocal vector of the diffracting planes of the unstrained crystals and the *x* axis is directed along 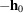 . Hence, as shown in Fig. 1 ▸, 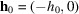 .

The sign of 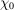 depends on the sign choice in the exponent of the plane wavefunctions. One can use either 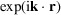 or 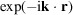 . In the former case, 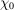 is positive, and in the latter case, it is negative.

The 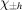 phases depend on the choice of the origin of the coordinate system in the unit cell; a translation 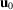 changes 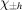 according to 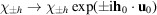 . We assume that, for the unstrained planes, 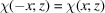 , so that 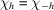 . Since 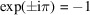 , the sign of 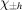 can be chosen as either plus or minus.

## 4. Crystal fields

We limit this study to crystals that are symmetrically cut and plane parallel. This choice makes the X-ray propagation two dimensional and dependent only on the inward normal 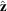 to the crystal surfaces and an *x* coordinate that we choose opposite the reciprocal vector 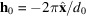 , where 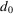 is the spacing of the diffracting planes of the unstrained crystals (Mana & Palmisano, 2004 ▸; Sasso *et al.*, 2022 ▸).

Owing to the limited spatial coherence of conventional X-ray sources, each incoming photon is in a probabilistic superposition of single-particle states 

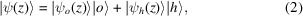

where we used the Dirac bra–ket notation and 

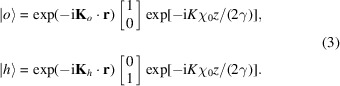

The 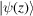 state belongs to the tensor product 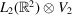 of the 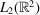 space of the square-integrable two-variable functions and the two-dimensional vector space 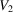 . Throughout the paper we use the 2 × 1 matrix representation of 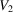 . Hence, by omitting the exponentials in (3), 

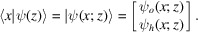

In (3), the mean electric susceptibility of silicon 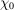 is set equal to zero in a vacuum. 

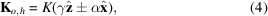

where 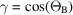 and 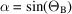 are direction cosines and 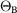 is the Bragg angle, are the kinematical wavevectors satisfying the Bragg conditions 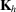 = 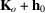 and 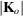 = 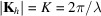 . We will use the subscript 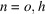 to label the 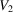 basis vectors and the first (plus or minus) sign of 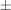 and 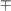 applies always to the *o* state. Also, we consider a coplanar geometry, that is, 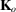 , 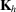 , 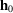 and 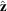 are in the same (reflection) plane.

The representation of the crystal fields as the components of a state vector (Bonse & Graeff, 1977 ▸) allows us to use matrix descriptions of optical components. This simplifies the study of the interferometer, the description of which can be built by assembling simpler elements. This approach is a useful alternative to the standard formulation of the dynamical theory of X-ray diffraction and an additional tool for the study of X-ray interferometry.

In this paper, we consider only the propagation of the coherent single-photon state (2). The averaging over their probabilistic superposition can be done by the density matrix formalism, as shown by Sasso *et al.* (2022 ▸).

## 5. Takagi–Taupin equations

The first-order approximation in 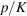 , where the *p* momentum is conjugate to *x*, of the X-ray propagation in a deformed crystal is given by the Takagi–Taupin equations (Takagi, 1962 ▸, 1969 ▸; Taupin, 1964 ▸; Katagawa & Kato, 1974 ▸; Authier, 2001 ▸; Härtwig, 2001 ▸; Mana & Montanari, 2003 ▸; Mana & Palmisano, 2004 ▸; Honkanen *et al.*, 2018 ▸), 

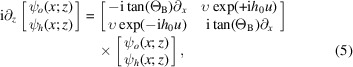

where 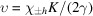 . We consider initial Gaussian-like beams and set the axis of the X-ray beam passing through the *x*-axis origin; therefore, at 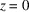 , 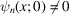 only if 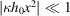 . The rationale for this assumption will be clear in the discussion following equation (10).

To solve the Takagi–Taupin equations, we factor 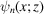 as (Mana & Palmisano, 2004 ▸) 

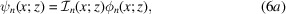

where, by setting 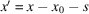 and 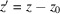 , 

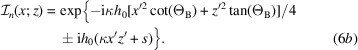

Therefore, (5) reads (see the supporting information) 

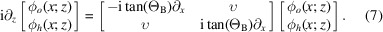




Now, it is convenient to use the Fourier transform of 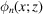 with respect to the *x* variable. Hence, 

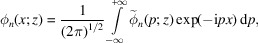

which leads to the reciprocal-space representation of the Takagi–Taupin equations, 

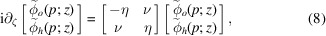

where 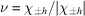 , 

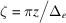

is the dimensionless propagation distance, 

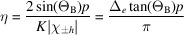

is the dimensionless resonance error, and 

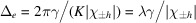

is the *Pendellösung* length.

Eventually, crystal propagation is given by 

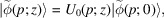

where, by solving (8) (see the supporting information), 

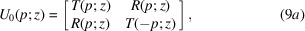




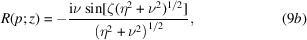




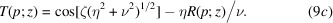




To complete the analysis, we need the 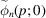 components of the initial state, which are obtained via the convolution integral 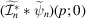 . Similarly, after propagation through a crystal having thickness *t*, we can retrieve the 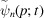 components of the output state via the convolution integral 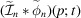 .

To calculate these convolution integrals, we rewrite (6*b* [Disp-formula fd6b]) as 

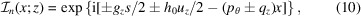

where we omitted inessential (constant) phases shared by the *o* and *h* states and a phase proportional to 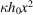 , 

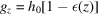

is the *x* component of the reciprocal vector 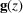 , 

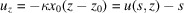

is the displacement field at 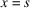 purged of the overall displacement *s*, 

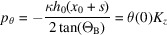

is the resonance error (Authier, 2001 ▸) that makes 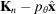 satisfy the Bragg condition at the hitting point 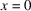 of the X-rays, and 

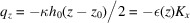

is the resonance error that makes 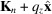 satisfy the Bragg condition versus the *x* component of the reciprocal vector 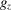 . The physical interpretations of 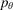 and 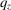 are given in the supporting information.

In (10), the omission of the 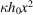 phase simplifies the convolution integrals, which otherwise must be approximated (giving the same result) by the steepest descent method. It is justified by assuming a limited transverse extension of the X-ray beam about 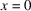 , *i.e.* 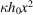 is assumed negligibly small everywhere 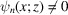 .

Note that 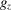 , 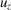 , 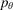 and 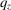 are independent of *x*. When examining the bending effect on the phase-contrast topography of a monolithic interferometer, we set *s* = 0. Therefore, 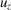 is the displacement field at *x* = 0, where the X-rays hit the crystal. When studying the bending effect on the measurement of the Si lattice parameter by a split-crystal interferometer, we set 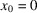 and 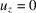 .

The Fourier transforms of (10) and of its complex conjugate are (see the supporting information) 

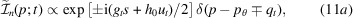




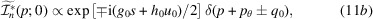

where the *g*, *u* and *q* subscripts 0 and *t* indicate 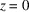 and 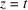 , *t* being the crystal thickness. The 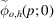 components of the initial state are given by the convolution integrals 

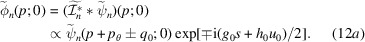

Similarly, the 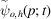 components of the final state are 

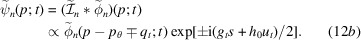

After ending the transformation chain describing the X-ray propagation through a bent crystal, 

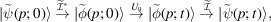

we observe that the result is the same as (see the supporting information) 

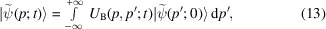

where

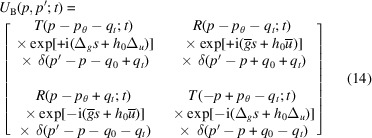

is the reciprocal-space representation of the propagator, 

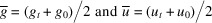

are, respectively, the averages of the reciprocal vector 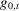 and displacement 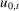 at the input (subscript zero) and output (subscript *t*) surfaces, and 

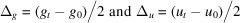

are their half differences.

As shown by (11*a* [Disp-formula fd11a]) and (11*b* [Disp-formula fd11b]), the 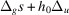 and 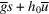 phases originate in the matching (ensuring the required continuity) of the input and output waves 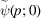 and 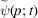 with the guided waves inside the crystal, *i.e.* the eigenmodes of the Hamiltonian of the Takagi–Taupin equations (5). For this reason, they depend on the lattice parameter and displacement fields at the crystal interfaces. In particular, X-ray propagation as given by (13) and (14) does not depend on the crystal displacement and lattice parameter inside the crystal.

It can be easily verified that, in the case of a displaced perfect crystal, *i.e.* 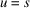 , the scattering matrix (14) reduces to (9*a* [Disp-formula fd9a]), where the reflection coefficient 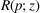 gets the 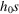 phase. This makes it possible to measure the spacing of the diffracting planes by making the *o* and *h* input states interfere.

If the strain is uniform, *i.e.* 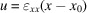 , then 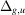 and 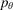 are equal to zero. Therefore, apart from the different Bragg angle encoded by the resonance error 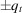 , the scattering matrix (14) reduces again to (9*a* [Disp-formula fd9a]), where the reflection coefficient gets the 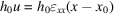 phase and the interference of the *o* and *h* input states yields a moiré pattern of upright fringes.

Eventually, if the deformation is a tilt of the diffracting planes, *i.e.* 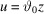 , then 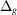 and 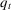 are equal to zero and 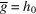 , 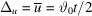 and 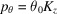 . Therefore, the scattering matrix (14) reduces to that given by Sasso *et al.* (2022 ▸) to account for a tilted crystal.

## 6. Free-space propagation

When studying the interferometer operation, the free-space propagation from one crystal to the next must also be considered. It is given by 

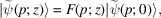

where the 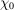 value in (3) must be set to zero and (see the supporting information) 

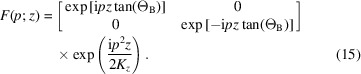

The first-order phase 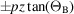 corresponds to geometric optics. Accordingly, the *o* and *h* states propagate in the 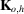 directions. Thus, we have 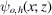 = 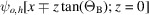 .

In contrast to propagation in crystals, we approximated the free-space propagation up to the order 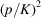 , which brings the 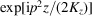 factor and recovers the 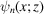 spread because of diffraction. This higher-order approximation is necessary to take into account the propagation of the different plane-wave components of the initial state. As we will make clear in the next section, it allows the incoming diverging rays, one of which is scattered in the 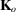 direction and the other in the 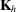 direction, to leave the source from different points.

## 7. Laue diffraction

**Figure 2:**
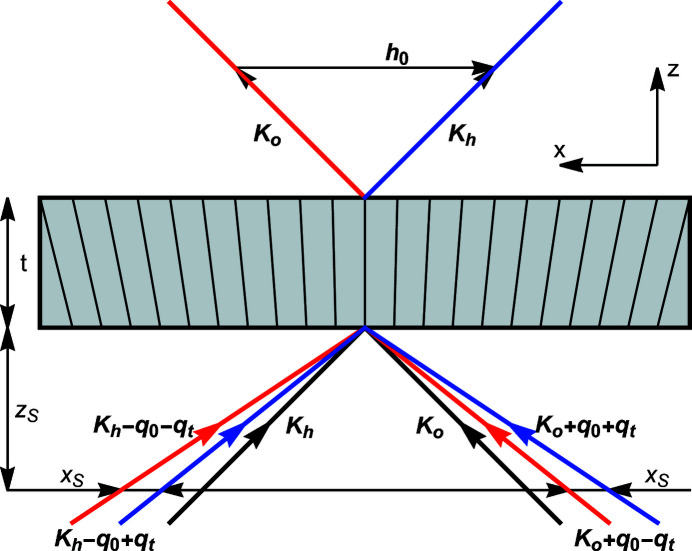
Laue diffraction by a bent crystal. 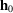 , reciprocal vector of the unstrained crystal; 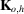 , diffracted kinematical wavevectors satisfying the Bragg law for the unstrained crystal; red and blue lines, incoming rays leaving the crystal in the 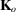 (red) and 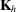 (blue) directions; black lines, rays incoming in the 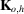 directions; 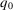 and 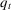 , resonance errors that make 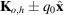 and 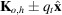 satisfy the Bragg condition versus the 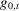 components of the reciprocal vectors at the input (subscript 0) and output (subscript *t*) surfaces, respectively; 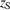 , source distance from the crystal; *t*, crystal thickness.

When X-rays, coming from a source at a distance 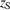 in the *o* or *h* state, impinge on a cylindrically bent crystal (plane parallel and symmetrically cut) as shown in Fig. 2 ▸, the waves leaving the crystal are (see the supporting information) 

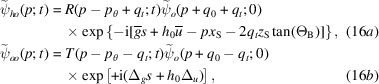

if the input state is 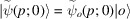 , and 

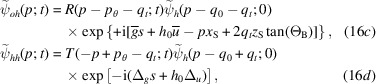

if the input state is 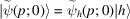 . They are given by (13), where 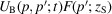 substitutes for 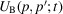 .

We omitted second-order terms proportional to 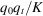 and irrelevant phases shared by the leaving waves, *t* is the crystal thickness, 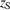 is the source distance, 

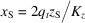

is the separation at the source of the rays that leave the crystal in the 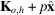 directions (see Fig. 2 ▸), 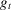 and 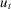 are, respectively, the reciprocal vector and displacement on the crystal exit surface and on the axis of the X-ray beam, and 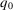 and 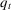 are the additional resonance errors on the crystal input and exit surfaces due to the crystal strain.

The 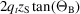 phase difference between the forward-transmitted and reflected waves originates in the free-space propagation of the rays exiting the crystal in the 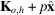 directions. In fact, they leave the source with different resonance errors, 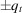 (see Fig. 2 ▸), and, thus, propagation directions.

The phases 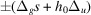 and 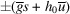 that come into the forward-transmitted and reflected waves play an essential role in the interferometer operation. As shown in the next section, according to how they add or subtract, they make the interference signal sensitive to the lattice parameter and displacement fields of one or the other side of the bent crystal.

## 8. Triple-Laue interferometer

The X-ray propagation through a triple-Laue interferometer having a bent crystal (the splitter or mirror or analyser) is given by 

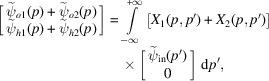

where 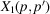 and 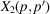 propagate 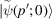 along the first and second arm of the interferometer, respectively. They are built by concatenating crystal and vacuum propagations.

The interferometer unstrained crystals have parallel and unshifted diffracting planes. Therefore, X-ray propagation is carried out by means of (9*a* [Disp-formula fd9a]). In contrast, propagation in the bent crystal is carried out by means of (14). Vacuum propagation is given by (15). Eventually, to examine separately the two interferometer arms, we introduce the projectors 

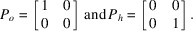

Free-space propagation leads to the separation of the *o* and *h* states, leaving the interferometer in two spatially localized states, whose 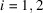 components overlap and interfere.

In the following subsections, we give the expressions of 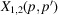 and 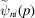 when the bent crystal is the splitter, mirror or analyser. In the 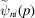 expressions, we neglect inessential phase terms shared by the interfering beams. The detailed calculations are given in the supporting information.

### 8.1. Splitter

When the bent crystal is the splitter, X-ray propagation along the two interferometer arms is given by 

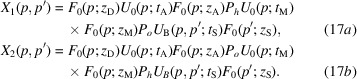

Fig. 1 ▸ gives the meaning of the symbols related to the interferometer geometry (crystal thicknesses and spacing, source and detector distances) that are used here and in the following subsections. The interfering waves reaching the detector are 

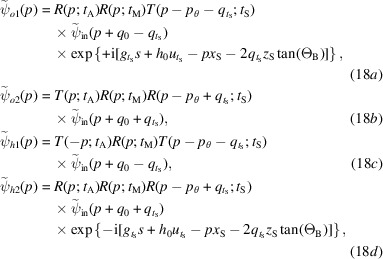

where 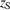 is the source distance from the splitter, 

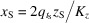

is the separation at the source of the rays interfering collinearly, 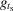 and 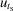 are, respectively, the reciprocal vector and displacement field on the splitter exit surface, and 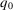 and 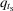 are evaluated on the splitter entrance (subscript 0) and exit (subscript 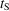 ) surfaces. As regards 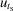 , it is evaluated on the axis of the X-ray beam.

Here and in the next subsections, we leave out the phase terms shared by the interfering wave pairs 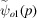 and 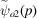 (*o* state) and 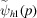 and 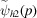 (*h* state). In addition, we assign the phase difference between the interfering waves to the wave reflected by the analyser, *i.e.* to 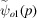 (*o* state) and 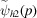 (*h* state), respectively.

The phases 

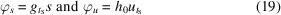

of the 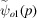 and 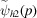 waves leaving the interferometer originate travelling fringes and moiré interference patterns that encode the diffracting-plane spacing 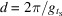 and displacement field 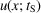 of the splitter inner surface 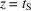 . In fact, according to equations (16*a* [Disp-formula fd16a])–(16*d* [Disp-formula fd16c]), the waves travelling along the 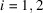 arms acquire, when crossing the splitter, the 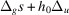 and 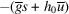 phases, respectively, whose difference is 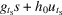 .

### 8.2. Mirror

When the bent crystal is the mirror, X-ray propagation is given by 

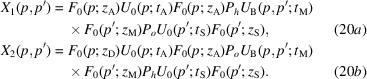

The detected waves are 

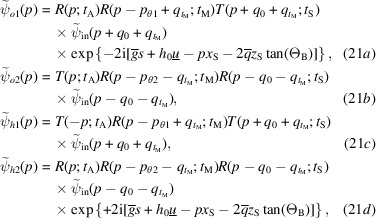

where 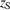 is the source distance from the mirror, 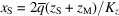 is the separation at the source of the rays interfering collinearly, 

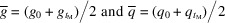

are, respectively, the means of the reciprocal vector and resonance error at the input (subscript 0) and output (subscript 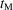 ) surfaces of the mirror, and 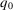 and 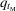 are evaluated on the mirror entrance and exit surfaces. As regards

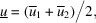

it is the average of the mean displacements 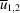 calculated along the first (subscript 1) and second (subscript 2) X-ray paths and on the beam axes. Since 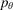 depends on the *x* coordinate along the mirror, the subscript *i* in 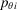 indicates the mirror crossing of the 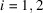 arms.

The phases 

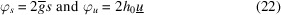

of the 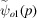 and 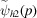 waves leaving the interferometer originate travelling fringes and moiré interference patterns that encode the means 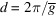 and 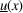 of the diffracting-plane spacing and displacement field, respectively, of the mirror input and output surfaces. In fact, according to equations (16*a* [Disp-formula fd16a])–(16*d* [Disp-formula fd16c]), the interfering waves, when crossing the mirror, acquire phases having identical 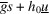 magnitude, but opposite signs.

### 8.3. Analyser

When the bent crystal is the analyser, the X-ray propagation is given by 

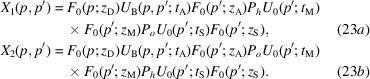

The interfering waves are 

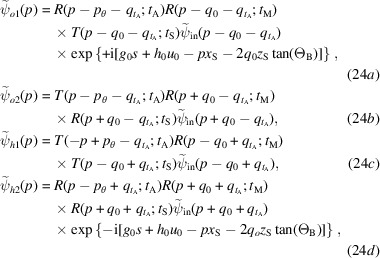

where 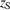 is the source distance from the analyser, 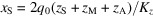 is the separation at the source of the rays interfering collinearly, 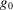 and 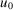 are the reciprocal vector and displacement field, respectively, on the input surface of the analyser, and 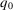 and 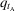 are evaluated on the input (subscript 0) and output (subscript 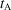 ) surfaces of the analyser. As regards 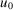 , it is evaluated on the axis of the X-ray beam.

The phases 

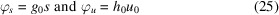

of the 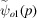 and 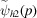 waves leaving the interferometer originate travelling fringes and moiré interference patterns that encode the diffracting-plane spacing 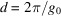 and displacement field 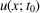 of the analyser inner surface 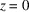 . In fact, according to equations (16*a* [Disp-formula fd16a])–(16*d* [Disp-formula fd16c]), the waves travelling along the 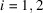 arms acquire, when crossing the analyser, the 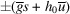 and 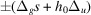 phases, respectively, where the plus (minus) sign applies to the leaving *o* 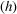 state. The phase difference is 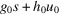 .

## 9. Conclusions

The terms 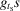 (if the displaced crystal is the splitter), 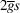 (if the displaced crystal is the mirror) and 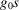 (if the displaced crystal is the analyser) in the phase difference of the waves travelling along the first and second arms [see (18[Disp-formula fd18a]), (21[Disp-formula fd21a]) and (24[Disp-formula fd24a])] make it possible to measure the diffracting-plane spacing. In the case of a displaced mirror, the period of the travelling fringes is half the spacing of the diffracting planes.

Our analysis of the interferometer operation confirms that, in the case of a bent analyser, the sought spacing is measured on the input surface. In fact, in equations (24[Disp-formula fd24a]), the observed phase difference is 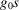 , where 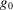 is the *x* component of the reciprocal vector at the input surface of the analyser. Supported by this result, we surmise that, if the measurement is repeated after flipping the analyser, a difference appears whenever the analyser is (smoothly) strained. These measurement repetitions were used to test the analyser’s perfection and corroborate the measurement results (Massa *et al.*, 2011 ▸, 2015 ▸).

The phase differences 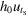 (splitter), 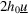 (mirror) and 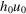 (analyser) [see (18[Disp-formula fd18a]), (21[Disp-formula fd21a]) and (24[Disp-formula fd24a])] are proportional to the displacement fields of the output surface of the splitter, 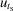 , the input surface of the analyser, 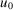 , and the mean 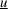 of the displacement fields of the two mirror surfaces. They made it possible to perform experimental tests of our results by the phase-contrast topography of a monolithic interferometer having the splitter or analyser bent by a Cu coating of one of its sides (Massa *et al.*, 2023 ▸). We predict that the interferogram is insensitive to what surface (input or output) of the mirror is coated. In contrast, we predict that it is sensitive to which surface (input or output) of the splitter or analyser is coated.

In equations (18[Disp-formula fd18a]), (21[Disp-formula fd21a]) and (24[Disp-formula fd24a]), the arguments of the reciprocal-space representations of the input wavefield 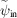 show that the rays interfering collinearly, *i.e.* having the same resonance error *p* when they leave the interferometer, exit the source with different resonance errors, 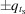 (if the bent crystal is the splitter), 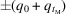 (if the bent crystal is the mirror) or 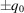 (if the bent crystal is the analyser). This is the same as saying that they leave the source at different angles. This difference implies two additional terms in the phase difference between the interfering waves.

The first, 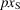 , encodes, via the time-shifting property of the Fourier transform, the fact that the rays interfering collinearly start from different points, spaced by 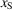 . This raises questions about the effect of the source coherence and suggests that a density matrix formalism is needed to describe the interferometer operation (Sasso *et al.*, 2022 ▸).

The second, 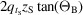 (if the bent crystal is the splitter), 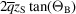 (if the bent crystal is the mirror) or 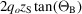 (if the bent crystal is the analyser), encodes the different free-space propagation from the source to the interferometer of the rays interfering collinearly. This difference is equal to zero in a perfect interferometer and we surmise it occurs whenever the crystals are (smoothly) strained. Since it makes the interference fringes sensitive to the source distance, a test of the interferometer sensitivity to it might additionally prove (or disprove) the crystals’ perfection and, if insensitive, certify the measured values of the diffracting-plane spacing.

Bending causes misalignment of the interferometer splitting and recombining crystals. Firstly, the misalignment stems from the difference between the lattice spacings of the strained and unstrained crystals. This difference is revealed via the 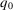 and 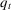 terms in the arguments of the reflection and transmission coefficients. It is independent of the crystal translation and X-ray incidence point – which, in (1), are encoded by the *s* and 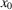 parameters – and originates a meaningless constant contribution to the fringe phase.

Secondly, the misalignment stems from the shear strain 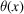 of the bent crystal. It is seen in the 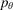 term in the argument of the reflection and transmission coefficients, which now depend on the X-ray incidence point. When scanning the X-ray incidence point, this misalignment mimics a continuous rotation of the crystal and it is equivalent to misalignments investigated by Mana & Vittone (1997*a* ▸,*b* ▸) and Sasso *et al.* (2022 ▸). The implied phase changes are very small in all practical cases.

## Supplementary Material

Mathematica notebook. DOI: 10.1107/S1600576723002844/ei5093sup1.txt

## Appendix A List of the main symbols

, normal to the diffracting plane.

, normal to the crystal surface.

, reciprocal vector (unstrained crystal).

, diffracting-plane spacing (unstrained).

, Bloch-wave wavevectors.

, Bragg law (unstrained crystals).

, Bragg angle (unstrained crystals).

,  ’s *z* direction cosine.

,  ’s *x* direction cosine.

, *z* component of  .

, Fourier components of the electric susceptibility.

.

, *Pendellösung* length.

, dimensionless resonance error.

, dimensionless propagation distance.

, crystal thicknesses.

, source and detector distances.

, start separation of the rays interfering collinearly.

, crystal displacement.

, reciprocal vector (unstrained crystals).

, reciprocal vectors (strained crystal, *x* components).

, resonance errors (normal strain).

, displacement fields.

, mean of the input and output surfaces.

, mean of the input and output surfaces.

, mean of the input and output surfaces.

, mean of the  paths.

, input–output difference.

, input–output difference.

, resonance error (shear strain).

, wavefield components (subscript).

, interferometer arm (subscript).

, crystal surfaces (subscript).

, the first sign applies to the *o* state, the second to the *h* one.
